# The clinical evaluation of IIA balloon occlusion in caesarean delivery for patients with PAS: a retrospective study

**DOI:** 10.1186/s12884-022-04434-3

**Published:** 2022-02-05

**Authors:** Ling Hong, Aner Chen, Jinliang Chen, Xiuxiu Li, Wenming Zhuang, Yijing Shen, Qiaohong Dai, Li Zhang

**Affiliations:** 1 Department of obstetrics and gynecology, Ningbo women and children’s hospital, Ningbo, China; 2 Radiology department, Ningbo women and children’s hospital, Ningbo, China; 3 Department of science and education, Ningbo women and children’s hospital, 339 liuting street, Ningbo city, Zhejiang province China

**Keywords:** Placenta accreta spectrum, Placenta accrete, IIA balloon occlusion

## Abstract

**Objective:**

The aim of this study is the clinical evaluation of IIA balloon occlusion in the caesarean delivery in patients with a diagnosis of placenta accreta spectrum.

**Background:**

High incidence of cesarean section leads to the increasing incidence of placenta accreta spectrum (PAS), which contributes to serious consequences such as severe obstetric postpartum hemorrhage or even maternal mortality.

**Methods:**

Fifty-eight patients with a diagnosis of PAS were retrospectively reviewed. The balloon group consisted of 23 patients, who underwent a caesarean delivery with internal iliac artery occlusion. 35 patients were in the control group, who had a standard caesarean delivery. The primary outcomes were estimated blood loss (EBL). The secondary outcomes were cesarean hysterectomy, blood transferring volume, operating time, intraoperative hemostatic approaches, surgical complications, balloon catheter–related complications, length of maternal stay, cost of hospitalization, and neonatal outcomes.

**Results:**

No difference was observed in estimated blood loss (EBL), blood transferring percentages and volume, additional measures to secure hemostasis, surgical complications, hospital stay postoperatively and newborn outcomes. More than 40% of the balloon group underwent hysterectomy because of uncontrollable postpartum bleeding (10 [43.48%] vs. 11 [31.43%], *P*=0.350). Complications related to occlusion of IIA did not occur. The duration of the surgery of the balloon group was significantly longer than that of the control group (123.52 min±74.76 versus 89.17±48.68, *P*=0.038), and the total hospitalization cost was also significantly higher than that of the control group (45116.67±9358.67 yuan versus 30615.41±11587.44 yuan, *P*=0.000).

**Conclusion:**

It does not permit to draw final conclusions for us on the effectiveness of the balloons IIA given the heterogeneity of selection of cases undergoing the procedures in the retrospective design. However, it is possible that IIA balloon occlusion may contribute to limiting intraoperative blood loss in more severe cases, particularly those undergoing peripartum hysterectomy.

## Background

The term placenta accreta spectrum disorder (PASD) is used to describe the morbidly adherent placenta, which includes placenta accreta, increta and percreta [1]. In past decades, women increasingly preferred to deliver by cesarean section (CS), leading to the increasing incidence of placenta accreta spectrum (PAS) [2]. It is reported with an incidence of 1 per 533 pregnancies [3]. One of the main and deadliest complications of PAS is massive bleeding. The average blood loss in these cases has been estimated to range between 2 and 3 liters [4]. Additionally, the morbidly adherent placenta is the most common indication for peripartum hysterectomy, which is associated with high rates of morbidity and mortality. Therefore, strategies to prevent and treat bleeding are are therefore critical. Various approaches have been employed in controlling intraoperative blood loss in cases of abnormal placentation, including inserting uterine tamponade balloons, applying uterine gauze packing, placing uterine brace or isthmic compression sutures, and performing a hysterectomy.

The arterial occlusion balloon has been used in PAS recently, and the role of internal iliac artery (IIA) balloon occlusion to improve hemorrhagic outcomes in women with placenta accreta has been evaluated in many studies, some of which showed benefit [5–9], but others failed to demonstrate any [10–12]. The aim of this study is the clinical evaluation of IIA balloon occlusion in the caesarean delivery in patients with a diagnosis of placenta accreta spectrum.

## Methods

Fifty-eight patients with a diagnosis of PAS underwent caesarean delivery between January 2015 and June 2018 at Ningbo Women and Children’s Hospital were retrospectively reviewed in this study. The Institutional Review Board of Ningbo Women and Children’s approved this study, and the need for informed consent was waived because of the retrospective nature of this study.

**Fig. 1:**
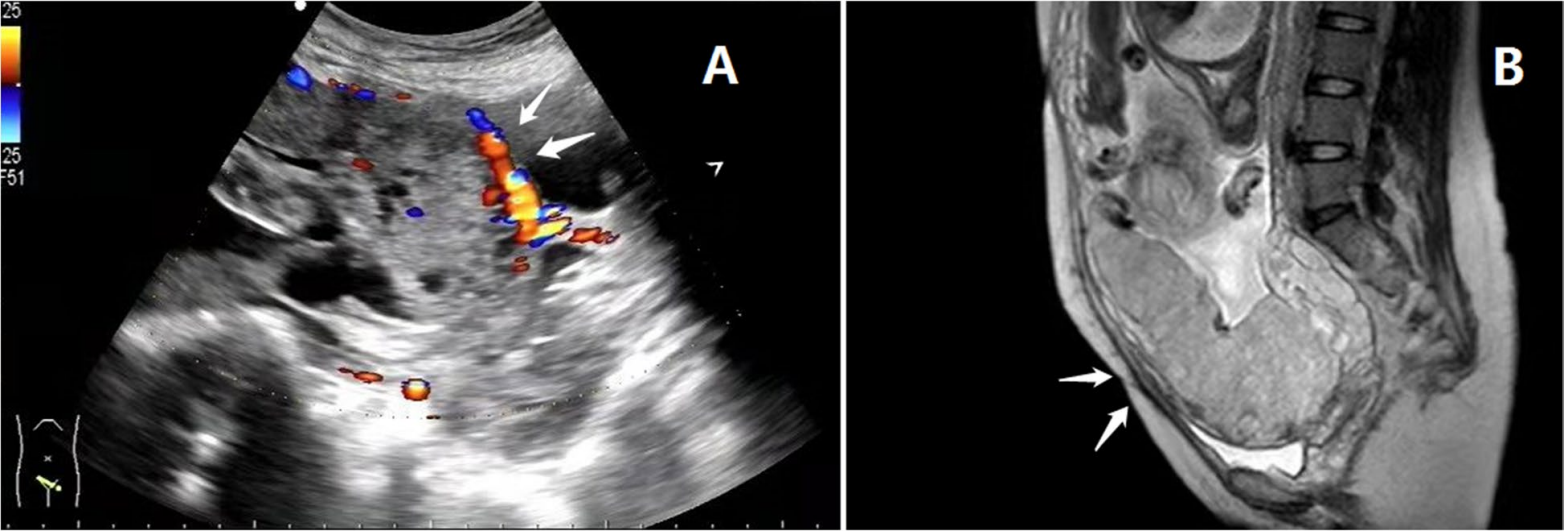
**A** Color Doppler images. The arrow indicates evidence of placenta accreta (indistinct boundary of utero-placental interface); **B** MRI. Placenta accreta in the lower uterine segment (indicated by an arrow)

Electronic medical records were reviewed to collect patient demographics, obstetrical history, operative reports, and clinical notes. The inclusion criteria were a diagnosis of PAS and having caesarean sections performed by the same experienced surgeon. For all women included in this study, a diagnosis of PAS had been made in prenatal imaging with either ultrasound and/or magnetic resonance imaging (Fig. 1) and abnormal placental status was confirmed clinically [1], or histopathologically, after delivery. The following patients were excluded: those who had a history of gynecological cancers or other solid cancers, hematological malignancies, gynecological surgeries (such as myomectomy, cervicoplasty and uterine rupture repair); those severe platelet disorders; twin pregnancy, those who underwent emergent delivery and cases with missing data. Each clinical case was discussed in multidisciplinary meetings with interventional radiologists, obstetricians, neonatologists, and urologist. The women who had consented to and undergone IIA balloon occlusion were assigned to the balloon group, while others were assigned to the control group. Patients who were not diagnosed antenatal but were diagnosed with placental implantation after delivery may also be included in the control group. Finally, there were 23 patients in the balloon group and 35 patients in the control group. Two women who underwent balloon occlusion but did not end up with a diagnosis of PAS were excluded. Occlusion balloon placement was performed by one of two senior interventional radiologists, who each had more than five years of experience with the similar procedure of uterine artery embolisation for caesarean scar ectopic pregnancy.

**Fig. 2:**
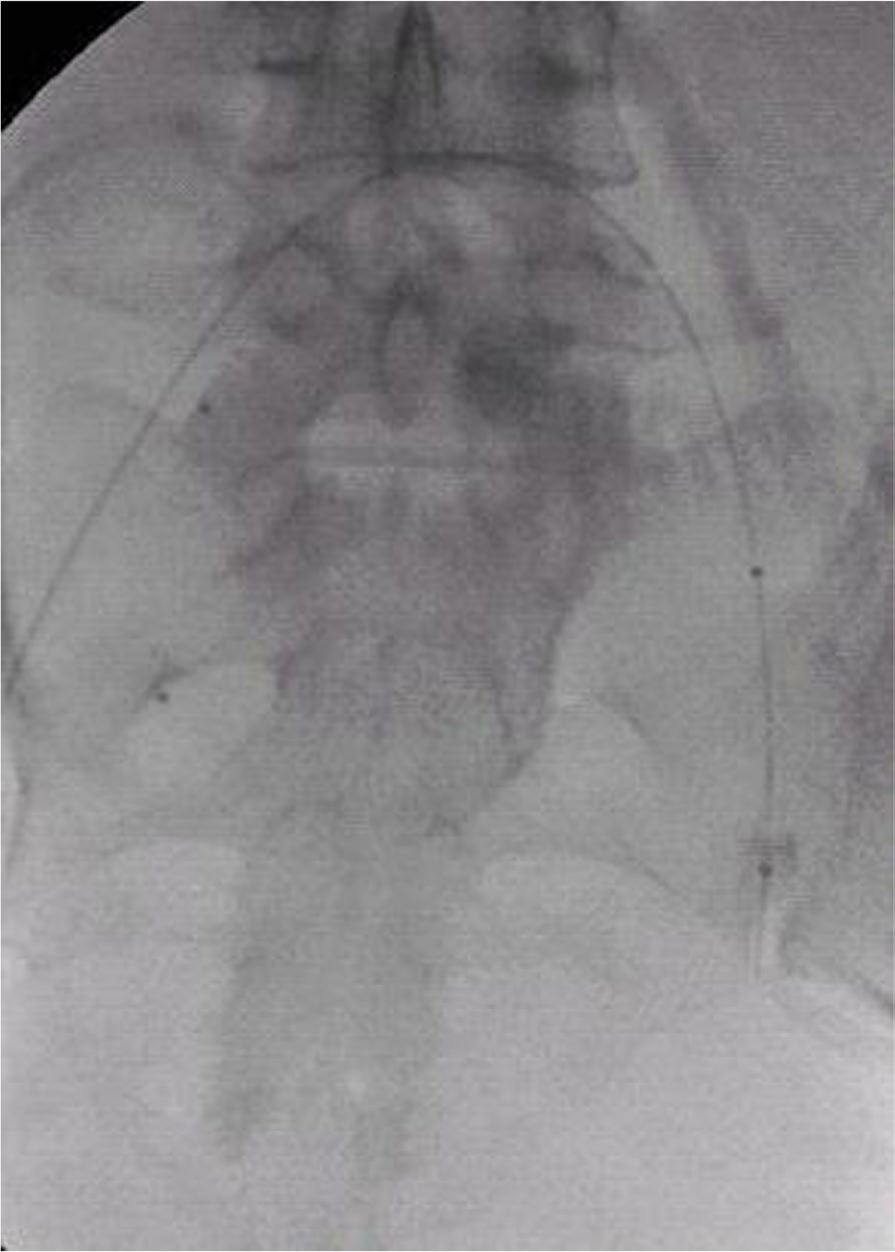
Fluoroscopy showing the balloon catheters positioned in the main lumen of the internal iliac arteries bilaterally

Procedure for the management of IIA balloon catheters was as follows. Arteriopuncture of bilateral femoral arteries via the Seldinger technique was performed after local anesthesia; 6- French sheaths were then put in place. Next, selective contralateral IIA catheterization was performed with fluoroscopy guidance, and a PTA catheter (Abbott, USA) was inserted into the IIA. The balloon (Abbott, USA), which was approximately 8-10 mm in diameter, was exchanged by using a 0.035- inch super- hard exchange guide wire and then positioned in the IIA with the head pointing to the branch of the anterior IIA, as confirmed by angiography (Fig. 2). After the insertion, The balloon was briefly inflated, and contrast injected to verify occlusion of the artery. Once positioning was satisfactory, the balloon catheter and its sheath were fixed on the skin. Patients were then transferred to the operating room for cesarean delivery. After the baby was delivered, the occlusion balloons were inflated immediately. The placenta was then delivered and cesarean delivery was continued in the usual manner. Balloons were inflated for approximately 30 minutes intraoperatively The balloon catheters were deflated after hemostasis was achieved, and 1 sheath was left in place for 12 hours to allow for resuscitation and emergency embolization if needed. The balloon was inflated or deflated by an interventional radiologist during the whole CS. Perioperative management was similar in the two groups except for catheter placement and balloon occlusion of bilateral internal iliac arteries.

The primary outcomes were estimated blood loss (EBL). The secondary outcomes were as follows: cesarean hysterectomy, blood transferring volume, operating time, intraoperative hemostatic approaches ( such as intrauterine tamponade, compression suturing, uterine artery ligation), surgical complications, balloon catheter–related complications (including puncture site hematoma, thrombosis, embolic events, and vascular rupture), length of maternal stay, cost of hospitalization, and neonatal outcomes. Statistics Statistical analysis was performed using SPSS 20.0 software. Data were presented as mean ± SD, median (range) or count (percentage). Comparison between 2 groups was determined by t test, Wilcoxon rank sum test or chi-square test. *p* < 0.05 was considered significant.

## Result

Fifty-eight women were included. Twenty- three women were in the balloon group and thirty-five women were in the control group. Baseline characteristics are summarized in Table 1. There was no significant difference between the two groups with respect to patient age at delivery, gestational age, gravidity, parity, type of pregnancy, number of previous uterine surgeries and abdominal surgeries, number of previous cesarean deliveries, types of placenta accretion and hemoglobin before cesarean section.

**Table 1:** Demographic characteristics and incidence of different forms of placenta accretion in the two study groups

Characteristic/Outcome	Balloon Group(*n*=23)	Control group(*n*=35)	*p-*value
Mean age, years	33.00±3.77	33.37±4.44	0.742
gestational age, weeks	34.86±1.05	35.47±1.20	0.113
Gravidity	3.70±1.11	3.91±1.29	0.508
Parity	2.13±0.55	2.14±0.43	0.924
Number of previous uterine surgeries	1.52±1.47	1.74±1.17	0.528
Number of previous cesarean deliveries	1.09±0.29	1.14±0.36	0.531
Hemoglobin before cesarean section, g/dl	11.23±1.33	11.19±1.25	0.914
Deepth of Placenta percreta			
Accreta	4	11	0.232
Increta	9(56.52%)	16(77.14%)	0.620
Percreta	10(43.48%)	8(22.86%)	0.097

The intraoperative and postoperative outcomes for the two groups are summarized in Tables 2 and 3. The median EBL was 2500ml (range, 1750-5750ml) in the balloon group versus 2000ml (range, 1500-3350ml) in the control group (*p*=0.171). There was no statistically significant difference observed between the groups with intraoperative hemorrhage volumes of 2500 mL and above (*p*=0.421). More than 40% of the balloon group underwent hysterectomy because of uncontrollable postpartum bleeding (10[43.48%] vs. 11[31.43%], *p*=0.350). The need for additional measures to secure hemostasis similarly showed no difference between the groups (Table 3).

**Table 2:** Blood loss and transfusion requirements between the two study groups

Parameters	Balloon Group(*n*=23)	Control group(*n*=35)	*p*-value
Estimated blood loss , ml^a^	2500(1750-5750)	2000(1500-3350)	0.226
Blood loss≥2500ml, n(%)	13(56.52%)	16(45.71%)	0.421
Transfusion, n(%)	21(91.30%)	35(100%)	0.076
Autologous blood transfusion, n(%)	20(86.96%)	34(97.14%)	0.134
Autologous blood transfusion, ml^a^	600(335-1050)	500(278.5-745.5)	0.347
RBC transfusion, n(%)	12(52.17%)	17(48.57%)	0.788
RBC, units^a^	3(0-6)	0(0-4.5)	0.486
FFP transfusion, n(%)	18(78.26%)	20(57.14%)	0.098
FFP, units^a^	1080(555-1200)	500(0-1160)	1.000
Cryoprecipitate transfusion, n(%)	12(52.17%)	10(28.57%)	0.070
Cryoprecipitate, units^a^	5(0-10)	0(0-9.75)	0.343

*RBC* Red blood cells, *FFP* Fresh frozen plasma

^a^Data were presented as median (range). Comparison between 2 groups was determined by Wilcoxon rank sum test

**Table 3:** Surgical details and postoperative outcomes between the two study groups

Parameters	Balloon Group(*n*=23)	Control group(*n*=35)	*P*-value
Cesarean hysterectomy, n(%)	10(43.48%)	11(31.43%)	0.350
Additional measures to secure hemostasis, n(%)	15(65.22%)	27(77.14%)	0.320
Iodoform gauze packing of the uterine cavity, n(%)	14(60.87%)	26(74.29%)	0.280
Balloon tamponade, n(%)	8(34.78%)	20(57.14%)	0.096
Uterine artery ligation, n(%)	2(8.70%)	2(5.71%)	0.661
uterine artery embolization, n(%)	6(26.09%)	12(34.29%)	0.509
Partial excision of the invaded uterine wall, n(%)	3	2	0.303
Duration of the surgery, min*	123.52±74.76	89.17±48.68	0.038
Hospital stay postoperatively, days	7.96±2.14	7.74±1.90	0.692
Hospitalization cost, yuan*	45116.67±9358.67	30615.41±11587.44	0.000
Surgical complications, n(%)	14(60.87%)	22(62.86%)	0.879
Fever, n(%)	11(47.83%)	20(57.14%)	0.487
DIC, n(%)	2(8.70%)	4(11.43%)	0.738
Pneumonedema, n(%)	0	1(2.86%)	0.414
Bladder injury, n(%)	3(13.04%)	1(2.86%)	0.134
Relaparotomy, n(%)	2(8.70%)	3(8.57%)	0.987
Maternal death	0	0	-
Balloon related complications			
Hematoma puncture	0	0	-
Pseudoaneurisms	0	0	-
Vein thrombosis	0	0	-
Neonatal outcome			
Neonatal birth weight, g	2540±480	2740±360	0.074
Apgar score of 1min	8.70±0.56	8.46±1.12	0.349
Apgar score of 5min	9.78±0.42	9.69±0.93	0.642
Neonatal mortality	0	0	-

DIC disseminated intravascular coagulation

*P<0.05

Twenty-one women in the balloon group and all women in the control group received blood transfusions. No significant differences were noted both in the percentages and volumes of autologous blood transfusion, red blood cell transfusion, fresh frozen plasma transfusion and cryoprecipitate transfusion between the two groups. There was no significant difference in the Hb decrease between the two groups (1.3 (range, -1.5-1.7) vs 1.35 (range, 0.1-2.45), *p*=0.266).

The duration of the surgery of the balloon group was significantly longer than that of the control group (123.52 min±74.76 versus 89.17±48.68, *p*=0.038), and the total hospitalization cost was also significantly higher than that of the control group (45116.67±9358.67 yuan versus 30615.41±11587.44 yuan, *p*=0.000). There was no significant difference in surgical complications, postoperative hospital stay, newborn weight, Apgar scores at 1 and 5 minutes between the two groups. No still births or maternal death was observed in either group. No balloon related complication occurred in the balloon group.

## Discussion

This study showed that balloon occlusion of the IIA in patients with PAS did not reduce the hysterectomy rate during a cesarean section, nor did it reduce blood loss and blood transfusion, but it prolonged the duration of the surgery and increased the total cost.

Although advances in obstetric care have led to a substantial improvement in pregnancy outcomes, the death rate from PAS remains as high as 7%, largely due to massive hemorrhage [13, 14], which may then lead to disseminated intravascular coagulation, fluid overload, acute respiratory distress syndrome and infection. It seems logical that occlusion of the internal iliac arteries with prophylactically- placed balloon catheters would be a more effective treatment option, but reported results are controversial. Some retrospective studies [6–9] reported that intraoperative IIA balloon occlusion had benefits in reducing blood loss and the amount of blood transfusion. Some previous systematic reviews also reported that intraoperative IIA balloon occlusion had benefits in reducing blood loss, the volumes of blood transfusion and even the percentages of cesarean hysterectomy in women with accrete [5, 15]. Nicholson et al. Also reported that patients with IIA balloon occlusion had a decreased rate of hysterectomy compared to those without it [16]. Recently, Yao FAN and Soo BuemCho 's two randomized controlled studies also agree with this view [15, 17]. However, randomized controlled studies [10, 18] and several case control studies [11, 12, 19] did not find any benefit. Our findings accord with the previous randomized controlled trial conducted by Meng Chen et al [18].

In our study, all patients are operated by the same surgeon, who has more than 20 years of experience and performs nearly 1,000 cesarean sections a year. They also received the same standard peripartum care apart from the insertion of iliac artery balloons which also eliminate the surgical procedural heterogeneity between surgeons and teams. This is the most significant advantage of our research, which has not been reported in other studies.

In our study, the two groups had similar blood loss, it might be possible that balloons contributed to limit blood loss of more severe cases (more percreta and increta in the IIA balloon group and more accreta in the control group, although not statistically significant), so we cannot exclude a role of the balloons in reducing blood loss in more severe cases. Given difficulties of prenatal diagnosis of PAS, should we consider positioning balloons only in suspected percretas? It might be worth digging into. On the other hand, collateral circulation of the ovarian artery and balloon displacement may also one of the reasons why occlusion of the internal iliac arteries failed to reduce hemorrhage [20, 21].

In our study, the transfusion rate was 91.3% in the balloon group and 100% in the control group, which was much higher than the 50% or so reported in the literature [22]. In our hospital, autologous blood transfusion is one of the necessary means to reduce the bleeding during the operation of pregnant women with placenta accreta spectrum. Therefore, nearly every pregnant woman in our study underwent autologous blood transfusion during a cesarean section, which may be the reason for the increased transfusion rate in our study.

Our results show that the duration of surgery in the balloon group is significantly longer than in the control group, which should be interpreted with caution. Pregnant women in the balloon group needed an interventional surgeon to remove the balloon after surgery, whereas women in the control group did not.

This study has several limitations, the most important of which was that the decision of balloon positioning was highly dependent on physician consultation with patients, and therefore there is the suspicion of a selection bias (balloon positioned in more severe cases). Then, we consider all the PAS equally, not differentiating grade 1,2 or 3. In the study, blood loss was estimated, and transfusion was performed based on surgeon preference, which may have varied based on practice.

## Conclusion

It does not permit to draw final conclusions for us on the effectiveness of the balloons IIA given the heterogeneity of selection of cases undergoing the procedures in the retrospective design. However, it is possible that IIA balloon occlusion may contribute to limiting intraoperative blood loss in more severe cases, particularly those undergoing peripartum hysterectomy.

## Data Availability

The research data used to support the findings of this study were supplied by Ms. Zhang under license and so cannot be made freely available. Requests for access to these data should be made to Ms. Zhang (Email:zhangli00501465@outlook.com).

## Abbreviations

PASD: Placenta accreta spectrum disorder
PAS: Placenta accreta spectrum
IIA: Internal iliac artery
EBL: Estimated blood loss
RBC: Red blood cells
FFP: Fresh frozen plasma
Hb: Haemoglobin concentration
DIC: Disseminated intravascular coagulation
